# Complete mitochondrial genome sequence of the Agaricomycetes brown rot fungus *Fomitopsis pinicola* isolate FBCC1181

**DOI:** 10.1128/MRA.00503-23

**Published:** 2023-11-01

**Authors:** Janina Österman-Udd, Taina Kristina Lundell

**Affiliations:** 1 Department of Microbiology, Faculty of Agriculture and Forestry, University of Helsinki, Helsinki, Finland; University of California, Riverside, California, USA

**Keywords:** mitogenome, mtDNA, basidiomycetes, *Fomitopsis*, endonuclease

## Abstract

The mitochondrial genome of the brown rot fungus *Fomitopsis pinicola* isolate FBCC 1181 is a 66.5 kbp circular chromosome. It contains 64 predicted genes, including a set typical for Basidiomycota Agaricomycetes mitogenomes. Introns of *cox* and *cob* genes contain several homing endonucleases of both LAGLIDADG and GIY-YIG types.

## ANNOUNCEMENT

Currently, there are 673 fungal mitochondrial genomes available in the organelles section of the NCBI Genome database (1), with only 54 belonging to Basidiomycota. The only mitochondrial genome available for *Fomitopsis* species is that of *Fomitopsis palustris* strain FFPRI 0507_CJ35 (2), found in the Nucleotide database (accession NC_034349.1). Here, we report the complete mitochondrial genome sequence for isolate FBCC 1181 of the *Fomitopsis* type species, *Fomitopsis pinicola* (Sv.) P. Karst, a wood-decaying brown rot fungus (3).

The mitochondrial genome (mitogenome) was assembled from long reads produced with PacBio Sequel II from total DNA, extracted from mycelium cultured in liquid malt extract medium, using a modified hot cetyltrimethylammonium bromide (CTAB) extraction protocol (4). A sequencing library was prepared using the SMRTbell Express Template Prep Kit 2.0 (PacBio). HiFi sequencing was performed on SMRTbell templates >10 kb, and raw data were processed with SMRTlink v11 to obtain HiFi reads. Fungal mitogenomes generally have a lower GC-content than the nuclear genomes (5) and a plot of the GC-values of all HiFi reads showed two groups of reads with distinct GC content, enabling the separation of mitochondrial reads from nuclear genomic reads. Reads with GC-content <40% longer than 10 kbp were selected using fastp v. 0.23.2 (6) and downsampled to 339× average depth of coverage (proportion 0.005) using Seqkit v. 2.3.0 (7). Assembly of the resulting 1,287 reads (22,586,710 bp; N50 = 17,564) using hifiasm v. 0.16.1-r375 (8) produced a primary assembly consisting of a single 97.65 kbp contig. However, the first and last 31 kbp of this contig were overlapping, resulting in a circular chromosome of 66,498 bp in size. Gene predictions and functional annotations were performed using MFannot (9, 10), and tRNA genes were further verified by tRNAscan-SE 2.0.11 (11). AGAT v1.0.0 (12) was used to transform the annotation information into gff3 format, followed by manual curation. The predicted mRNA sequences were extracted using gffread v0.12.7 (13) and verified using NCBI blast (https://blast.ncbi.nlm.nih.gov/Blast.cgi) (14). Predicted open reading frames were subjected to Batch CD-Search (15) to determine the presence of homing endonucleases.

**Fig 1:**
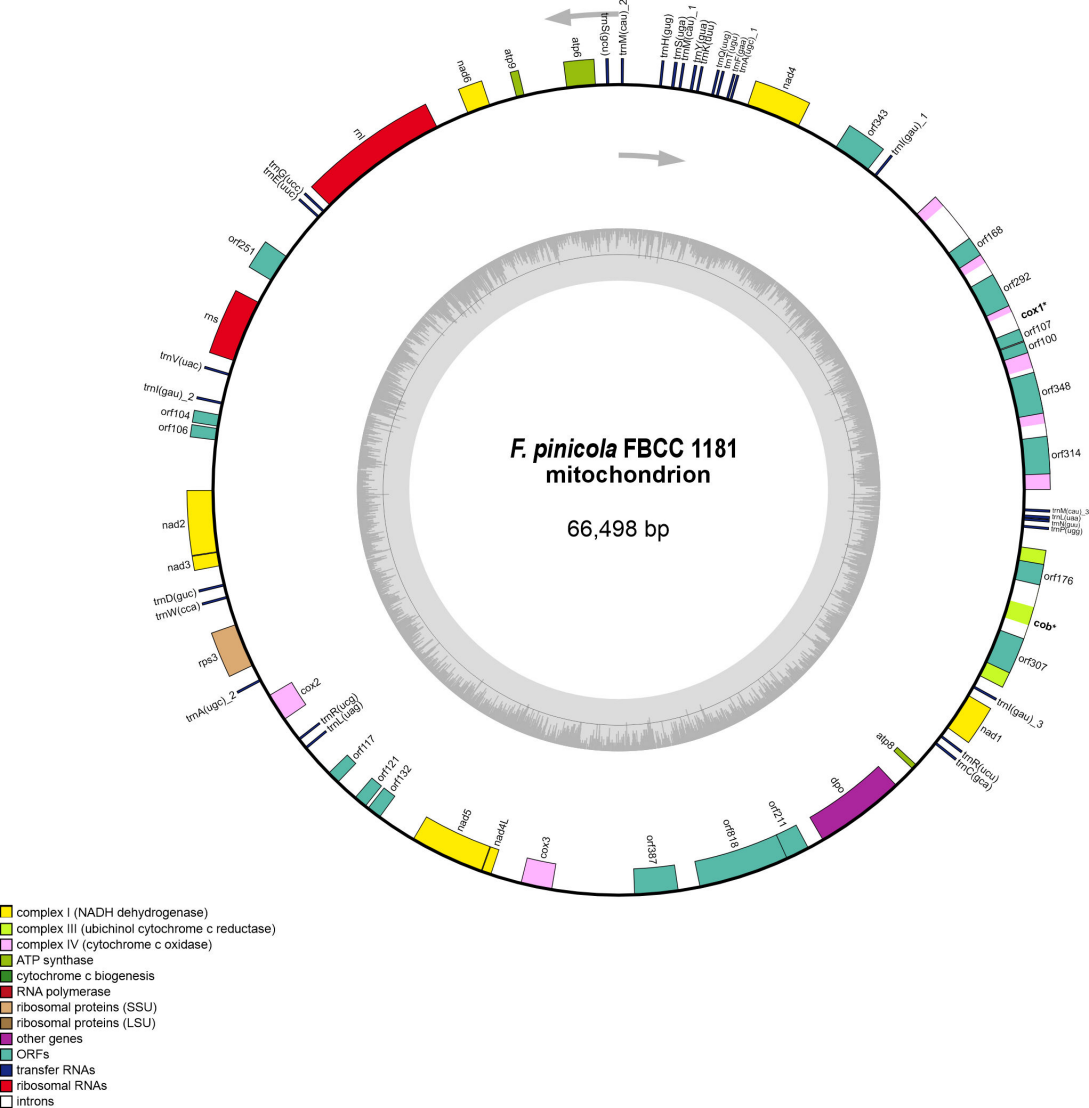
Graphical representation of the mitochondrial genome of *F. pinicola* FBCC 1181, produced with OGDRAW (16) and edited with CorelDRAW v. 24.1.0.360.

The average size of all currently available Basidiomycota mitochondrial genomes is 78 kbp and typically contains 14 conserved protein-coding genes, two ribosomal RNA genes, and 20–31 tRNA genes (2, 4, 5). In addition, many Basidiomycota mitogenomes contain genes for ribosomal protein S3 (*rps3*) and DNA polymerase (*dpo*) (2, 4). The mitogenome of *F. pinicola* strain FBCC 1181 is 66.5 kbp in size with an average GC-content of 22.6% and displays all 14 conserved protein-coding genes for oxidative phosphorylation: cytochrome *c* oxidase subunits (*cox*1–3), apocytochrome b (*cob*), NADH dehydrogenase subunits (*nad1–6* and *nad4L*), and ATP synthase subunits (*atp6, atp8*, and *atp9*; Fig. 1). In addition, the protein-coding genes *rps3* and *dpo* are present, as well as the ribosomal RNA genes *rnl* and *rns*. There are 28 tRNA genes for the 20 common amino acids. Four LAGLIDADG and two putative GIY-YIG endonuclease genes were identified within introns of the *cox1* and *cob* genes.

## Data Availability

The complete mitochondrial genome sequence with gene annotation has been deposited at the European Nucleotide Archive (ENA) and is available under BioProject accession PRJEB58965. Raw reads used for genome assembly are available under ENA accession ERR11083882. This presentation is the first version.
